# Supplementary material for: Effectiveness of a Self-Decontaminating Coating Containing Usnic Acid in Reducing Environmental Microbial Load in Tertiary-Care Hospitals
Source: Int J Environ Res Public Health. 2023 Apr 7;20(8):5434. doi: 10.3390/ijerph20085434 (PMC10138069; doi:10.3390/ijerph20085434)
Supplement: Supplementary file 1 [file ijerph-20-05434-s001.zip › ijerph-2295689-supplementary.pdf]

Table S1. Identified bacteria and fungi in Hospital A by sampling site and sampling phase.

No	Sampling site*	Sampling 1** (CFU/10^2 cm^2)	Sampling 2** (CFU/10^2 cm^2)	Sampling 3** (CFU/10^2 cm^2)	Sampling 4** (CFU/10^2 cm^2)
1	wall	*Corynobacterium* sp (1)<br>CNS (3)	no growth	no growth	no growth
2	examination bed	no growth	no growth	no growth	no growth
3	trolley	CNS (10)<br>*Aspergillus niger* (1)	no growth	no growth	no growth
4	counter	*Corynobacterium* sp (6)	no growth	no growth	no growth
5	wall	CNS (3)<br>*Aspergillus niger* (1)	no growth	no growth	no growth
6	wall	no growth	no growth	no growth	no growth
7	examination bed	CNS (3)	no growth	no growth	no growth
8	trolley	CNS (3)	no growth	no growth	no growth
9	examination bed	*Staphylococcus aureus* (1)	no growth	no growth	no growth
10	trolley	no growth	no growth	no growth	no growth
11	waiting room seat	*Corynobacterium* sp (3)<br>CNS (15)<br>*Aspergillus niger* (2)	no growth	no growth	no growth
12	admission counter	*Staphylococcus aureus* (8)<br>CNS (4)<br>*Micrococcus* sp (2)	no growth	no growth	no growth
13	waiting room seat	*Corynobacterium* sp(1)	no growth	no growth	no growth
14	patients' counter	CNS (19)<br>*Micrococcus* sp (8)<br>*Corynobacterium* sp (2)<br>*Escherichia coli* (4)	no growth	no growth	no growth
15	waiting room seat	*Corynobacterium* sp (1)<br>CNS (2)	no growth	no growth	no growth
16	wall	*Staphylococcus aureus* (1)	no growth	no growth	no growth
17	wall	no growth	no growth	no growth	no growth
18	seat	no growth	no growth	no growth	no growth
19	seat	CNS (3)<br>*Micrococcus* sp (2)<br>*Corynobacterium* sp (1)	no growth	no growth	no growth
20	bed	CNS (2)	no growth	no growth	no growth
21	trolley	CNS (1)	no growth	no growth	no growth
22	trolley	*Staphylococcus aureus* (1)	no growth	no growth	no growth
23	bedside cabinet	CNS (6)<br>*Corynobacterium* sp (1)	no growth	no growth	no growth
24	wall	no growth	no growth	no growth	no growth
25	bed	CNS (15)<br>*Corynobacterium* sp (6)<br>*Candida albicans* (3)	CNS (1)	no growth	no growth
26	table	CNS (10)	no growth	no growth	no growth
27	nurse counter	*Staphylococcus aureus* (12)	no growth	no growth	no growth
28	wall	no growth	no growth	*Micrococcus* sp (1)	no growth
29	bed	no growth	no growth	no growth	no growth
30	table	*Staphylococcus aureus* (1)	no growth	no growth	no growth

		Candida albicans (3)			
31	trolley	no growth	no growth	no growth	no growth
32	trolley	*Staphylococcus aureus* (6)<br>*Corynobacterium* sp (2)	no growth	no growth	no growth
33	trolley	CNS (14)<br>*Escherichia coli* (2)	no growth	no growth	no growth
34	cabinet	no growth	no growth	CNS (1)	no growth
35	cabinet	CNS (1)	no growth	no growth	no growth
36	cabinet	no growth	CNS (1)<br>*Micrococcus* sp (2)	no growth	no growth
37	cabinet	CNS (1)<br>*Micrococcus* sp (2)	no growth	no growth	no growth
38	table	no growth	no growth	no growth	no growth
39	bed	*Staphylococcus aureus* (74)<br>*Candida albicans* (2)	no growth	no growth	no growth
40	bed	CNS (22)<br>*Escherichia coli* (2)	no growth	no growth	*Micrococcus* sp (1)
41	trolley	CNS (58)	no growth	no growth	no growth
42	wall	no growth	no growth	no growth	no growth
43	wall	CNS (16)<br>*Corynobacterium* sp (2)	no growth	no growth	no growth
44	wall	no growth	no growth	no growth	no growth
45	bed	CNS (54)<br>*Corynobacterium* sp (2)	no growth	no growth	no growth
46	CT controller	*Staphylococcus aureus* (6)<br>CNS (3)	CNS (24)<br>*Micrococcus* sp (4)	no growth	no growth
47	waiting room seat	*Staphylococcus aureus* (11)<br>*Corynobacterium* sp (2)<br>*Aspergillus fumigatus* (2)	no growth	no growth	no growth
48	wall	*Staphylococcus aureus* (2)	no growth	no growth	no growth
49	bed	CNS (22)<br>*Micrococcus* sp (10)<br>*Corynobacterium* sp (5)	no growth	no growth	no growth
50	workstation surface	CNS (3)	no growth	no growth	no growth
51	wall	*Micrococcus* sp (1)	no growth	no growth	no growth
52	bed	no growth	no growth	no growth	no growth
53	angiography trolley	*Staphylococcus aureus* (3)<br>CNS (3)	no growth	no growth	no growth
54	waiting room seat	CNS (1)	no growth	no growth	no growth
55	waiting room seat	CNS (4)<br>*Staphylococcus aureus* (1)	CNS (74)	no growth	no growth
56	stairs handrail	CNS (7)	no growth	no growth	no growth
57	stairs handrail	CNS (25)	no growth	no growth	no growth
58	stairs handrail	*Staphylococcus aureus* (6)<br>*Corynobacterium* sp (5)<br>*Escherichia* coli (2)	no growth	no growth	no growth
59	stairs handrail	no growth	no growth	no growth	no growth
60	stairs handrail	*Corynobacterium* sp (14)<br>*Aspergillus niger* (2)	no growth	no growth	no growth
61	stairs handrail	*Corynobacterium* sp (20)	no growth	no growth	no growth

		CNS (12)			
62	stairs handrail	CNS (10)<br>*Candida albicans* (1)	no growth	no growth	no growth
63	stairs handrail	CNS (10)	no growth	no growth	no growth
64	elevator button	*Staphylococcus aureus* (20)<br>CNS (15)<br>*Micrococcus* sp (5)	no growth	CNS (3)	no growth
65	elevator wall	CNS (47)<br>*Micrococcus* sp (20)	no growth	no growth	no growth
66	elevator door	CNS (3)	no growth	no growth	no growth
67	elevator button	CNS (5)	no growth	no growth	no growth
68	elevator wall	CNS (6)	no growth	no growth	no growth
69	elevator door	CNS (44)<br>*Micrococcus* sp (22)<br>*Corynobacterium* sp (1)	no growth	no growth	no growth

CFU: colony-forming unit; CNS: coagulase-negative Staphylococcus; ICU: intensive care unit; CT: computed tomography. *Samples 1-15: Emergency Department; samples 16-23: COVID-19 patients' rooms; samples 24-43: ICU; samples 44-55: Radiology and CT. Department; samples 56-69: stairs and elevators. **sampling 1: 9 days before the coating was applied; sampling 2: 3 days after the coating was applied; sampling 3: 10 days after the coating was applied; sampling 4: 21 days after the coating was applied.
